# Psoriatic arthritis: Pathogenesis and novel immunomodulatory approaches to treatment

**DOI:** 10.1186/1476-8518-3-6

**Published:** 2005-09-02

**Authors:** Sarah Cassell, Arthur Kavanaugh

**Affiliations:** 1 Center for Innovative Therapy, Division of Rheumatology, Allergy, and Immunology, The University of California, San Diego, 9500 Gilman Drive, La Jolla, CA 92093-0943, USA

## Abstract

Psoriatic arthritis (PsA) is a chronic inflammatory arthropathy characterized by the association of arthritis and psoriasis. PsA runs a variable course, from mild synovitis to severe, progressive, erosive arthropathy. The pathogenesis of PsA involves alteration in the components of the immune response, although the exact cause of PsA is unknown. A number of patients with severe peripheral arthritis fail to respond to standard conventional therapy. Advances in biotechnology and in our understanding of the immunopathogenesis of PsA have led to great interest and progress in regards to biologic treatments for PsA. Notable success achieved with recently introduced biologic therapies has paved the way for further research and develpoment of additional therapies that should improve outcomes for affected patients.

## Introduction

Psoriatic arthritis (PsA) is a chronic inflammatory arthropathy characterized by the association of arthritis and psoriasis. Joint involvement is heterogeneous, and may consist of spondyloarthropathy, as well as oligoarticular and polyarticular peripheral arthritis. PsA runs a variable course, from mild synovitis to severe, progressive, erosive arthropathy. PsA is classified as one of the subtypes of spondyloarthropathy, sharing clinical features such as asymmetric joint involvement, an oligoarticular arthritis pattern, a similar frequency in men and women, the common occurrence of enthesitis and dactylitis, infrequent rheumatoid factor and anti-cyclic-citrullinated-peptide seropositivity, and extra-articular manifestations such as iritis.

## Epidemiology

Psoriasis occurs in about 2% of the population [1]. PsA has been reported in 7% to 42% of patients with psoriasis [2]. The prevalence of PsA in the US has been estimated as 0.67% [3]. However, estimates of prevalence are variable, due in part to the heterogeneity of the disease as well as a lack of validated diagnostic criteria [4].

In general, skin involvement precedes joint disease, often by years. However, PsA precedes skin psoriasis in about 15% of patients, and the two occur simultaneously in about 20%. Some reports suggest that PsA is more common in patients with severe psoriasis [5,6]. A recent study suggested a correlation between the extent of skin and joint severity only among patients with simultaneous onset of skin and joint manifestations [7].

## Pathogenesis

The exact cause of PsA is unknown, although genetic, environmental, and immunologic factors clearly play important roles. The pathogenic connection between psoriasis and arthritis is not clear, although both are immunologically mediated.

### Genetic factors

Most studies document a familial predisposition to both psoriasis and PsA. More than 40% of patients with PsA have first degree family members with either skin or joint disease [8,9]. Several genetic susceptibility loci have been proposed, with the strongest effect residing within the major histocompatibility complex (MHC). Population studies in PsA have shown increased frequency of HLA-B13, B17, B27, B38, B39, DR4 and DR7 [8,10,11]. In a comparison of 158 patients with PsA to 101 patients with uncomplicated psoriasis, HLA-B7 and B27 were more common among patients with PsA, whereas B17, Cw6 and DR7 were more common among patients with uncomplicated psoriasis [8]. Some of these associations may be confounded by linkage disequilibrium. HLA-B27 has been associated with spinal disease in which radiological sacroiliitis is present. A symmetric pattern of peripheral PsA appears related to HLA-DR4 [8,12]. The strongest susceptibility locus for psoriasis is on chromosome 6p, termed PSORS1 [1,13-16]. Other psoriasis susceptibility loci are located on chromosomes 17q25 (PSORS2), 4q34 (PSORS3), 1q (PSORS4), 3q21 (PSORS5), 19p13 (PSORS6), 1p (PSORS7), and 17q25 (RUNX1) [1].

Other genes within the MHC region and non-HLA associations have been explored. A TNF-α promoter polymorphism or a gene in linkage disequilibrium with TNF-α may predispose or increase susceptibility to psoriasis and PsA [17]. One study looking at the TNFβ+252 and TNFα-308 polymorphisms did not find the alleles more frequently in PsA patients than matched controls, but did find both alleles were significantly associated with the presence of joint erosions and the progression of joint erosions in early PsA [18]. A meta-analysis showed the TNFα-238 variant in Caucasian PsA patients was a significant risk factor for PsA [19]. A recent study pointed to Cw6 and MHC class I chain-related A (MICA)-A9 as being the strongest genetic susceptibility factors for PsA [20].

### Environmental factors – infection, trauma

Both viral and bacterial infections have been implicated as causative agents in PsA. Support for the role of bacterial antigens in the pathogenesis of psoriasis and PsA comes from indirect observation of enhanced humoral and cellular immunity to gram-positive bacteria typically found in the psoriatic plaques [21]. However, psoriatic plaques often get secondarily infected, thus the cause-effect relationship of bacteria and psoriasis is difficult to prove. One study of sera from patients with PsA showed higher levels of antibody to streptococcal exotoxin, which provides some evidence of a link between streptococcal infection and articular inflammation [22]. The possibility that PsA might be virally induced has been proposed, although never confirmed [23,24]. Physical trauma may result in the onset of psoriasis (Koebner Phenomenon) and, theoretically, PsA at the sites of injury. This association would highlight potential association between innate and specific immunity. There are a number of case reports suggesting a possible role for trauma in PsA, but this has not been evaluated in a prospective manner.

### Immunologic factors

Both psoriasis and PsA are immunologically mediated. Characteristic pathologic features of PsA are synovial lining layer cell hyperplasia, inflammatory cell accumulation and prominent vascularity. T lymphocytes, particularly CD8+ cells, may play important pathogenic roles. Activated T cells have been noted in affected tissue, both skin and joint [25,26]. A predominance of CD8+ T lymphocytes with clonal expansion have been found in PsA synovial fluid leading to the proposal that CD8+ T cells drive the immune response [27]. This is further supported by the fact that CD8+ T cells also dominate the infiltrate at marrow sites adjacent to entheseal inflammation, an early area of involvement [28]. An analysis of T cell receptor beta chain variable (TCRβV) gene repertoires revealed common expansions in both skin and synovial inflammatory sites, suggesting an important role for cognate T cell responses in the pathogenesis of PsA and that the inciting antigen may be identical or homologous between afflicted skin and synovium [29].

The cytokine network in the psoriatic skin and synovium is dominated by monocyte and T-cell derived cytokines: IL-1β, IL-2, IL-10, IFN-γ and TNF-α [30]. In PsA synovium, higher levels of IFN-γ, IL-2 and IL-10 have been detected than in psoriatic skin. One study of cytokine staining in PsA synovium showed IL-1α, IL-1β, IL-8, IL-15, IFN-γ and TNF-α staining localized to the lining layer and perivascular macrophages [31]. These cytokines can induce proliferation and activation of synovial and epidermal fibroblasts, leading to fibrosis in patients with longstanding PsA. TNF-α, a key proinflammatory cytokine, induces the production of other inflammatory cytokines such as IL-1, IL-6, and granulocyte-macrophage colony-stimulating factor, chemokines such as IL-6, degradative enzymes such as several matrix metalloproteinases (MMPs) and other factors. TNF-α mediates a number of biological processes that can result in joint damage including stimulation of bone resorption, inhibition of bone formation, and inhibition of synthesis of proteoglycans [32,33]. Angiogenic factors such as TNF-α and vascular endothelial growth factor (VEGF) may contribute to vascular proliferation [34,35].

While the mechanisms governing psoriatic skin and joint involvement are similar, there are distinctions. For example, cutaneous lymphocyte associated (CLA) antigen, an adhesion molecule that identifies lymphocytes that preferentially traffic to the skin, is upregulated on lymphocytes in psoriatic skin but is minimally expressed on cells in the PsA synovium [36].

## Clinical Features

Wright and Moll recognized several patterns of PsA: isolated distal interphalangeal disease, peripheral oligoarthritis, peripheral polyarthritis, and spondyloarthropathy. These clinical phenotypes are not fixed but are interchangeable, and individual patients can switch phenotypes [37]. The most important distinction as regards outcome appears to be oligo- versus poly-articular joint involvement.

Extra-articular manifestations of PsA are important aspects of the disease, the most common is the psoriatic skin lesion, which may affect all areas of the skin. Dactylitis is typical in PsA and presents as inflammation of the whole digit, joints and tendon sheaths. Enthesitis, inflammation at the site of tendon, ligament or synovial membrane insertion into bone, is characteristic of PsA and may represent the earliest site of involvement. Other extra-articular manifestations include the presence of iritis, mouth ulcers, and urethritis.

PsA has several characteristic radiographic features which include lack of periarticular osteopenia, destruction of interphalangeal joints with widening of the joint spaces, pencil-in-cup changes in the hands and feet, ankylosis, periosteal reaction, and spur formation [38].

The course of PsA is variable. Patients who have five or more involved joints at presentation are more likely to have progressive disease. Some patients have few episodes and completely recover, but recent studies demonstrated that many patients have persistent and severe courses [39-41]. Damage in PsA occurs early and progresses over time, with increasing deformities and limitation of daily activity [42]. Patients with PsA have increased mortality compared to the general population. More severe disease, as manifested by higher ESR and radiologic scores at presentation, is a predictive factor of mortality [43].

## Treatment of PsA

### Conventional treatment

Mild joint symptoms may respond to non-steroidal anti-inflammatory drugs (NSAIDs) [42]. Systemic steroids can be used, but may cause side effects and rebound worsening of psoriasis [44]. Patients who are unresponsive to NSAID therapy or who have progressive disease may require disease modifying anti-rheumatic drugs (DMARDs) (eg methotrexate [MTX], leflunomide, sulfasalazine [SSZ], cyclosporine [CsA]).

MTX is considered by many rheumatologists the DMARD of choice because of its remarkable efficacy in ameliorating both skin and joint disease, its rapid onset, and its acceptable safety profile [45,46]. However, 16–30% of patients treated with MTX discontinue it because of toxicity [47,48]. Leflunomide, an antipyrimidine drug that interferes with T-cell activation, has been shown to be effective in improving both joint and skin symptoms [49]. The most common side effects seen with leflunomide are diarrhea and increased transaminases. SSZ has been shown to be helpful for peripheral arthritis but not for axial disease [50]. CSA improves both joint inflammation and skin lesions in PsA, but is not frequently used because of its toxicities, the most worrisome being hypertension and nephrotoxicity [48,51]. Likewise, gold compounds and other drugs have been reported to ameliorate arthritis in some PsA patients, but are rarely used secondary to side effects and toxicities.

### Biologic Agents

In recent years, greater understanding of immunopathology and advances in biotechnology facilitating the ability to design and produce novel biologic agents have led to exciting breakthroughs in the treatment of autoimmune disease, including psoriasis and PsA [52]. The development of novel biologic agents has been further encouraged by the unmet need for better treatments and the positive results with their use in other autoimmune diseases, particularly rheumatoid arthritis (RA). The most significant experience of the use of biologics in treatment of PsA is with TNF-α inhibitors.

#### Tumor necrosis factor-alpha (TNF-α) inhibitors

Given its pro-inflammatory potential and its elevated levels in RA and PsA, TNF-α was identified as an attractive target for biologic therapies. TNF-α inhibitors have been used with great success to suppress joint inflammation in RA, inducing not only marked improvement in the signs and symptoms of disease, but also substantially improved functional status and quality of life [53-55]. Additionally, they have been shown to attenuate the progression of radiographic joint damage. Adverse effects have been reported, but in general these agents are well-tolerated. These encouraging results spurred interest in using TNF-α inhibitors in PsA. Currently there are three TNF-α inhibitors available: 1) etanercept, a fusion protein consisting of a dimer of the extracellular portion of the type II TNF receptor (p75) linked to the Fc portion of IgG1, 2) infliximab, a chimeric monoclonal antibody specific for TNF-α, and 3) adalumimab, a human monoclonal antibody specific for TNF-α.

##### 1. Etanercept

Etanercept has been proven effective for the treatment of PsA [56,57]. The first double-blind, placebo controlled clinical trial of etanercept in PsA was in 60 patients with long-standing disease. The etanercept group showed significant improvement in all measures of disease activity compared with the placebo group at 12 weeks. The primary endpoint for arthritis activity, the Psoriatic Arthritis Response Criteria (PsARC), a composite index, was achieved by 87% versus 23% of the etanercept and placebo groups respectively [58]. A secondary endpoint was the American College of Rheumatology composite response criteria (ACR), a score based on 20%, 50%, or 70% improvement [59]. ACR20 responses were 73% and 13% in the etanercept and placebo groups respectively [56]. For psoriasis, the primary endpoint was 75% improvement in the Psoriasis Area and Severity (PASI) score (PASI75). Of patients with >3% body surface involvement, 26% of etanercept treated patients achieved PASI75 versus none in the placebo treated group [56]. Disability, as assessed by responses on the health assessment questionnaire (HAQ), significantly improved in the etanercept group. An open-label extension of this study revealed sustained efficacy in joints, further improvement of skin disease, ability to decrease or discontinue concomitant methotrexate and prednisone, and continued tolerability [60].

Another phase III clinical trial of etanercept in 205 patients with PsA confirmed and extended earlier findings. ACR20 response rates were achieved by 59% of the etanercept group and 15% of the placebo group at 12 weeks (P < 0.001). This clinical response was sustained for 24 weeks. Of those meeting criteria for PASI evaluation, the etanercept group showed on average 47% improvement compared to no improvement in the placebo group (P < 0.001) [57].

Etanercept has been observed to slow and halt radiographic structural damage in PsA. A one year study of 205 patients revealed that at twelve months the radiographic disease progression in the etanercept group was inhibited (Sharp score: -.03 units) compared with worsening in the placebo group (Sharp score: +1.00 units) (p = .0001) [61].

##### 2. Infliximab

Open-label studies of infliximab in PsA showed significant decreases in the signs and symptoms of joint inflammation and skin disease [62-64]. This led to double blind, placebo controlled trials, which also revealed positive results [65,66]. The infliximab multinational psoriatic arthritis controlled trial (IMPACT) enrolled 104 patients in a double blind, randomized, placebo-controlled trial for 16 weeks, followed by blinded single-crossover design through 50 weeks [65]. ACR20/50/70 responses at week 16 were 69%/49%/29% in the active treatment group compared to 8%/0%/0% in the placebo group. These results were sustained at 50 weeks with ACR 20/50/70 responses in the infliximab group of 72%/54%/35%. Of the placebo-treated patients who crossed over to active treatment at week 16, ACR20/50/70 responses increased to 77%/49%/30%. This study also assessed dactylitis and enthesitis, two important characteristics of PsA that had not previously been included in clinical trials. Significant improvements were seen in dactylitis and enthesitis with infliximab therapy. Of particular note in this study was the dramatic improvement in skin psoriasis seen with infliximab treatment. Thus, PASI75 was achieved by 12 of 14 infliximab patients whereas there was overall worsening of skin scores in the placebo treated group. This effect was sustained at week 50. Also, 8 of 16 placebo patients who switched to infliximab treatment after week 16 achieved PASI75 at week 50.

These results were confirmed with the subsequent larger phase 3 IMPACT 2 study [67]. In this trial, 200 patients with active PsA were randomized to receive infliximab or placebo for 24 weeks. ACR 20/50/70 scores at week 24 in the infliximab group were 54%/41%/27% and 11%/4%/2% in the placebo group. Again, skin improvement was very impressive, with 60% of the infliximab group achieving PASI75 at week 24, whereas only 1% of the placebo group did. Statistically significant improvements in measures of functional status and quality of life (measured by HAQ and SF-36, respectively) were seen, as were improvements in dactylitis and enthesopathy.

Two studies have shown that infliximab can inhibit radiographic disease progression. In a double-blind, placebo controlled trial of 200 PsA patients (IMPACT2), patients treated with infliximab had significantly less radiographic disease progression at week 24, as measured by the van der Heijde-Sharp method modified for PsA (-0.7 +/- 2.53 versus .82 +/- 2.62, for infliximab versus placebo treated patients respectively; p < 0.001) [68]. An analysis of patients from the IMPACT1 study showed that at 50 weeks, radiographic progression of disease was inhibited in both the group treated with infliximab throughout the trial as well as in the group receiving infliximab from week 16 through week 50 [69].

##### 3. Adalumimab

Adalumimab was assessed in PsA in a phase III, placebo-controlled, double blind study, the Adalimumab Effectiveness in PsA Trial (ADEPT) [70]. 151 patient received adalumimab and 162 received placebo. Adalumimab treated patients showed rapid improvements. At week 24 ACR20, 50, and 70 scores for adalumimab were 57%, 39%, and 23% respectively versus 15%, 6%, and 1% for placebo. PASI50, 75 and 90 scores for adalumimab and placebo respectively were 75%, 59%, and 42% versus 12%, 1%, and 0% [70].

Adalimumab was also shown to inhibit radiographic disease progression. In the ADEPT trial, at week 24 mean change in modified total Sharp Scores (mTSS) was -0.2 in infliximab treated patients compared with +1.0 in placebo treated patients (p <= .001). All patients were allowed to go into an open label extension after week 24. Patients who started in the placebo arm and crossed to the adalimumab open label arm at week 24 had mTSS scores of +1.0 and +1.0 at weeks 24 and 48 respectively, showing no further radiographic progression after they started adalimumab. The patients originally in the adalimumab arm who extended into open label treatment had mTSS scores of -0.2 and 0.1 at weeks 24 and 48 respectively. Assessments at week 48 showed that adalimumab maintained the lack of radiographic change [71].

With all TNF-α inhibitors there have been concerns about safety issues, particularly infections, serious infections and opportunistic infections such as reactivation of latent tuberculosis. Appropriate monitoring for signs and symptoms of infection is required before and during treatment. While other adverse events have been reported at relatively low rates, careful monitoring of patients on these new biologic agents is quite important.

#### Alefacept

Another biologic agent in development for PsA is alefacept, which was approved in the US for the treatment of psoriasis in 2003. Alefacept is a human LFA-3/IgG1 fusion protein and is under clinical investigation for the treatment of PsA and RA. The LFA-3 portion of alefacept binds to CD2 receptors on T cells to block the natural interaction between LFA-3 on antigen-presenting cells and CD2 on T cells. Blockade of the LFA-3/CD2 interaction, a key co-stimulatory pathway, can inhibit T-cell activation. The IgG1 portion of alefacept can bind to FcγRIII (CD16) IgG receptors on accessory cells (e.g. natural killer cells) and may induce granzyme-mediated apoptosis [52,72].

Alefacept was evaluated as a treatment for psoriasis in multicenter, randomized, placebo-controlled, double blind study. Two hundred twenty-nine patients with chronic psoriasis received intravenous injection of alefacept at different dosages. The mean reduction in the PASI score 12 weeks after treatment was greater in the alefacept groups than the placebo group [73].

A small study suggested that alefacept may also improve both skin and joint symptoms in PsA [74]. In a single center open-label study, 11 patients with PsA received intravenous 7.5 mg alefacept once weekly for 12 weeks. Synovial tissue biopsies of an index joint were obtained by arthroscopy at baseline and at weeks 4 and 12. Clinically, some degree of improvement in arthritis was observed in six patients (55%) at the completion of the treatment. A similar proportion of patients achieved 50% amelioration of skin disease. This study supports the notion that T cell activation plays an important role in chronic inflammatory diseases and effective blockade of the LFA-3/CD2 interaction may be useful for treating PsA.

Additionally, a double blind, placebo controlled trial assessed the combination of alefacept and methotrexate in 185 PsA patients. An ACR20 response was achieved by 54% of the alefacept group versus 24% of the placebo group. 53% of the alefacept group achieved PASI50 compared with 17% of the placebo group [75]. Adverse events in this trial occurring at >5% included: back pain, nasopharyngitis, nausea, URI and increased ALT. There were no serious infections and the serious adverse event rate was 2% [76].

#### Efalizumab

Leukocyte function associate antigen-1 (LFA-1) is an adhesion molecule expressed on T lymphocytes. It interacts with its ligand, intercellular adhesion molecule (ICAM-1), in ways that may be relevant to the pathogenesis of psoriasis including: stabilizing the binding of antigen-presenting cells to T lymphocytes, facilitating migration of T lymphocytes from circulation into skin, and activation of T lymphocytes [77]. Efalizumab is a humanized monoclonal IgG antibody that binds to the alpha-subunit (CD11) of LFA-1 and prevents LFA-1 binding to ICAM-1. In two recent phase 3, randomized, double-bind, placebo-controlled trials, efaluzimab showed efficacy in treating moderate to severe plaque psoriasis, and was recently approved for this use by the US FDA.

Leonardi et al, reported a study of 498 psoriasis patients that showed PASI75 scores at 12 weeks in the treatment groups were achieved in 32.6% of patients versus 2.4% of placebo-treated patients [77]. The most common adverse events (headache, fever, chills, nausea, and myalgias) were more frequent in the efalizumab-treated group only during the first two injections, and then decreased to rates similar to placebo. A second study randomized 556 psoriasis patients for twelve weeks with continuation in an open label study [78]. At 12 weeks, PASI50/75 were 58.5%/26.6% respectively in efaluzimab-treated patients compared with 13.9%/4.3% in placebo treated patients. These numbers increased at week 24. Patient reported outcomes (dermatology life quality index and itching scale) also improved. Interestingly, during the second twelve weeks there was an increased incidence of arthritis (5.6%); 12 of these 19 cases had had a prior history of arthritis.

Preliminary results from a phase II study of efalizumab in 117 PsA patients showed that treatment did not reach statistical significance as far as achieving an ACR20 reponse at twelve weeks [79].

#### Other types of biologic agents and future directions

The introduction of TNF-α inhibitors and their tremendous clinical impact has generated considerable interest in exploring other avenues for the treatment of PsA. In addition, it is worth noting that despite the tremendous success achieved in PsA patients treated with TNF-α inhibitors, approximately one-third of patients with moderate to severe PsA have negligible or insufficient responses to such treatment. This has provided the impetus for the development of biologic agents targeting other aspects of the dysregulated immune system. Several promising biologic agents, directed at targets other than TNF-α, are currently under study (Table 1).

**Table 1:** Biologic agents under consideration for the treatment of Psoriatic arthritis

**Suppression of inflammatory mediators**		
**target**	**agent**	**comment**
IL-1	Anakinra	IL-1 receptor antagonist
IL-8	ABXIL-8	human anti-IL-8 mAb
**Modulation of the function of Anti-inflammatory mediators**		
**target**	**agent**	**comment**
IL-10	rIL-10	recombinant human Th2 cytokine
IL-11	rIL-11	recombinant human Th2 cytokine
Alteration of T cell number and function interaction		
**target**	**agent**	**comment**
IL-12	anti-IL-12 mAb	several in development
CD25 (IL-2 receptor)	Daclizumab	humanized anti-CD25 mAb
CD2	Alefacept	human LFA-3/IgG fusion protein
CD11a (LFA-1)	Efalizumab	humanized anti-CD11a mAb
TCR/CD3	huOKT3γ1(ala-ala)	humanized anti-CD3 mAb
CD80/CD86	IDEC-114	humanized anti-CD80 mAb
	CTLA4Ig	fusion protein of CTLA-4/Ig
CD40/CD40L	IDEC-131	humanized anti-CD154 mAb

mAb, monoclonal antibody; rIL, recombinant interleukin; IL-2R, LFA, leukocyte function associated antigen; TCR, T-cell receptor; CTLA4Ig, cytotoxic T-lymphocyte-associated antigen 4/immunoglobulin

One approach is the targeting of other inflammatory mediators. ABXIL-8 (Abgenix Inc, Fremont, CA), a human anti-IL-8 monoclonal antibody, binds free IL-8 and may deactivate it in the skin. Effects of IL-8 include T cell and neutrophil activation and chemotaxis, as well as keratinocyte proliferation. IL-8 may also play a role in the vascular responses found in psoriasis [80]. A phase II trial in psoriasis showed some improvement in patients' PASI as well as in histological responses [81]. IL-1, many of the activities of which overlap with TNF, has been suggested to be of potential importance in the pathogenesis of joint and other inflammation [82]. Anakinra (IL-1ra) a homologue of the naturally occurring IL-1 receptor antagonist, has been approved for use in moderate to severely active RA. Other IL-1 inhibiting agents are in development. To date there have not been controlled clinical trials of IL-1 inhibitors in PsA.

Another approach that would suppress inflammation involves the therapeutic use of anti-inflammatory cytokines. For example, among its various activities, Il-10 inhibits INF-γ and promotes TH2 biased cytokine secretion. IL-10 is relatively deficient in psoriatic skin, although it is found in high levels in synovium and serum of PsA patients [83]. Recombinant IL-10 (rIL-10) was used in a phase II trial in 14 patients with chronic plaque psoriasis; 71% had more than a 50% reduction of PASI scores [84]. It has also been studied in PsA which showed modest improvements in skin but not articular disease [85]. Recombinant human IL-11 (rhIL-11) has been shown to have anti-inflammatory activity in vitro and in vivo and has been tested in 12 patients with psoriasis. They showed some improvement in PASI scores [86]. However, there are no published reports of it being used in PsA.

Another therapeutic strategy is to target the number or function of immunocompetent cells central to the propagation of the disease. Several therapies have targeted T cells, which have been suggested to play a central role in orchestrating the immune driven inflammation in PsA. Daclizumab, a humanized antibody to the α-subunit of the IL-2 receptor, blocks the binding of IL-2, a vital growth factor for T cells. One trial in 19 psoriatic patients showed IL-2 blockade during the first 4 weeks and variable desaturation after that, which correlated with reversal in disease improvement that had been achieved. Patients with pretreatment PASI score of <36 showed mean reduction in severity by 30% at eight weeks [87]. HuOKT3γ1 (ala-ala), a non-FcR-binding monoclonal antibody to CD3 (a component of the T cell receptor complex), modulates the function of T cells without decreasing their numbers. A phase I/II study in seven patients with PsA showed 6/7 achieving ACR70 responses at 30 days and all seven had transient, dose dependent depletion of T cells [88]. CD28 is a cell-surface protein on mature T cells and binds to two ligands, CD80 and CD86 on antigen-presenting cells. Blocking this interaction results in incomplete T cell activation. CTLA-4, a natural inhibitor of CD28, binds to CD80/86 molecules with high affinity and competes with CD28. CTLA-4-immunoglobulin (CTLA4Ig) was developed to block the CD 28 and CD80/86 interactions. A phase 1 trial in psoriasis patients showed dose dependent improvement in skin involvement [89]. A CTLA-4-Ig construct, abatacept, is in late phase development for the treatment of RA. It will likely be studied in PsA in the near future. IDEC-114, a humanized anti-CD80 monoclonal antibody, has also been developed to block this interaction. A phase I/II trial of IDEC-114 in 35 psoriatic patients showed 40% of patients achieved PASI50 [90]. Finally, therapies directed at inhibiting IL-12, a cytokine central in driving Th1 biased immune responses, are in the early phases of investigation in psoriasis and PsA.

## Conclusion

Appreciation of the unmet clinical need for affected patients, greater understanding of the underlying immunopathophysiology of this common autoimmune disease, and progress in biopharmaceutical development have paved the way for the development of novel biologic agents for PsA. Following closely upon the successes achieved in RA, there have been dramatic clinical efficacy achieved with TNF inhibitors. Substantial improvements have been noted not only as far as the signs and symptoms of arthritis, but also in dactylitis and enthesitis and in skin involvement. Moreover, improvements in functional status and quality of life, and attenuation in the progression of radiographic damage have been achieved. Driven by this success, biologic agents targeting other components of the dysregulated immune response that play major roles in pathogenesis of PsA are actively under study. In the foreseeable future, we can expect further exciting development in immunomodulatory therapies for psoriatic arthritis.
